# Amniotic Fluid Bacterial Colonization, Antibiotic Susceptibility, and Associated Factors Among Women With Premature Rupture of Membranes at Mbarara Regional Referral Hospital, Southwestern Uganda

**DOI:** 10.7759/cureus.72219

**Published:** 2024-10-23

**Authors:** Brenda Ainomugisha, Richard Migisha, Collins Agaba, Leevan Tibaijuka, Asiphas Owaraganise, Joy Muhumuza, Joel Bazira, Musa Kayondo, Joseph Ngonzi

**Affiliations:** 1 Obstetrics and Gynecology, Mbarara University of Science and Technology, Mbarara, UGA; 2 Physiology, Mbarara University of Science and Technology, Mbarara, UGA; 3 Clinical Division, Infectious Diseases Research Collaboration, Kampala, UGA; 4 Microbiology, Mbarara University of Science and Technology, Mbarara, UGA

**Keywords:** amniotic fluid, antibiotic susceptibility, bacterial colonization, premature rupture of membranes, uganda

## Abstract

Background: Amniotic fluid bacterial colonization in premature rupture of membranes (PROM) is known to initiate labor and lead to postpartum endometritis and early-onset neonatal sepsis. We determined the prevalence and factors associated with amniotic fluid bacterial colonization, described bacterial isolates and examined antibiotic susceptibility patterns among women seeking care at Mbarara Regional Referral Hospital (MRRH) in southwestern Uganda.

Methods: We conducted a cross-sectional study from December 21, 2020 to June 12, 2021. We consecutively enrolled women with PROM at ≥24 weeks of gestation and aseptically picked two endo-cervical swabs. Aerobic cultures were performed on blood, chocolate, MacConkey agars, and polymerase chain reaction on culture-negative samples. Antibiotic susceptibility was performed via the Kirby-Bauer disk diffusion and dilution method. Interviewer-administered questionnaires were used to obtain participants’ sociodemographic, medical, and obstetric characteristics. We performed multivariable logistic regression to determine factors associated with bacterial colonization.

Results: We enrolled 144 participants with a mean age of 26.5±6.2 years. The prevalence of cervical amniotic bacterial colonization was 35.4% (n=51; 95% confidence interval (CI): 28.0-43.7). Six bacteria were isolated: *Klebsiella pneumoniae*, *Staphylococcus aureus*, *Enterobacter agglomerans*, *Escherichia coli*, *Streptococcus spp.*, and *Enterococcus faecalis*. Ciprofloxacin exhibited the highest sensitivity (88.6%), followed by cefuroxime (75%), while all isolated bacteria demonstrated resistance to ampicillin. Factors independently associated with cervical amniotic fluid bacterial colonization were prime gravidity (aOR=2.69; 95%CI: 1.07-6.71,* p*=0.035), obesity (aOR=3.15; 95%CI: 1.10-9.11,* p*=0.024), and being referred-in (aOR=2.37; 95% CI: 1.04-5.3,* p*=0.038).

Conclusion: Approximately one-third of the women had amniotic fluid bacterial colonization, and this was associated with being prime gravida, being obese, and being referred. The most common bacteria isolated was *K. pneumoniae,* followed by *S. aureus*. There was good sensitivity to quinolones and cephalosporins, and all bacterial isolates were resistant to ampicillin - the recommended first line of treatment for PROM by the Ministry of Health calls for revision of guidelines.

## Introduction

Premature rupture of membranes (PROM) refers to a spontaneous disruption in the integrity of the amniotic sac with leakage of amniotic fluid after 24 weeks of gestation but before the onset of labor [1]. It is a common obstetric condition occurring in 10% of pregnancies globally and causing significant maternal and prenatal morbidity and mortality [2]. Ascending bacteria from the lower genital tract have also been implicated in the causation of PROM following infection and inflammation of the fetal membranes and placenta [3]. The prevalence of bacteria colonization in the cervical amniotic fluid varies between 20% and 50%, influenced by geographical region and the specific method employed to detect the presence of bacteria [4]. A study conducted at Mulago National Referral Hospital in Uganda revealed a prevalence of 30% for bacteria colonization in the cervical amniotic fluid [5].

Culture is considered the gold standard for determining bacterial colonization; however, some bacteria that have been implicated in the causation of PROM, such as *Urea urealyticum*, Mycoplasma species, *Fusobacterium nucleatum*, and Leptotrichia species, are uncultivable or difficult to cultivate [6]. Molecular-based identification techniques, such as the polymerase chain reaction (PCR), prove invaluable in cases where traditional cultivation methods are inadequate [7]. Amniotic fluid bacterial colonization in women with PROM has also been reported to be associated with several factors, including duration of PROM, prior antibiotic use, gestation age, obesity, urinary tract infections, and abnormal vaginal discharge [8].

Group B streptococcus (GBS), a common bacterium found in the vagina and rectum of women, has long been associated with the causation or complication of most cases of preterm PROM [9]. In response, Uganda's Ministry of Health adopted the World Health Organization's (WHO) recommendation of intravenous ampicillin along with oral erythromycin for the initial 48 hours, followed by oral amoxicillin or erythromycin for five days, as a prophylactic treatment for PROM [10]. However, recent studies conducted in Asia and Africa have indicated a shift in bacterial patterns in the amniotic fluid of women with PROM, with a decrease in GBS predominance and an increase in the prevalence of *Escherichia coli*, *Klebsiella pneumoniae*, and *Staphylococcus aureus*; furthermore, there is growing evidence of antibiotic resistance among these bacteria in women with PROM [11].

Data on bacterial isolates and antibiotic susceptibility patterns in Uganda, particularly in southwestern Uganda, are limited, yet antibiotics are routinely administered to mothers with PROM in Uganda. Thus, continued use of the current antibiotic guidelines in PROM without periodically reviewing the antibiotic resistance profile may not only increase resistance through the emergence of multidrug-resistant strains but also in drug waste and complications for patients due to ineffective treatment. This study determined the prevalence and associated factors of amniotic fluid bacterial colonization and described the bacterial isolates and antibiotic susceptibility patterns among pregnant women seeking care at Mbarara Regional Referral Hospital (MRRH) in Southwestern Uganda.

There is a preprint of this manuscript in Research Square: https://assets-eu.researchsquare.com/files/rs-3138651/v1/50fd994f-e817-4608-b1fa-4f5437f83a47.pdf?c=1691863647. This is because when I first submitted the manuscript to BMC Pregnancy and Childbirth, the preprint was posted on research square. The manuscript was eventually not published by BMC Pregnancy and Childbirth. However, the preprint still exists in the research square.

## Materials and methods

Study design and setting

A cross-sectional study was conducted at the maternity ward of MRRH from December 21, 2020, to June 12, 2021. MRRH is a public facility and a teaching hospital for Mbarara University of Science and Technology (MUST) located in Southwestern Uganda. It serves as a referral center for over ten districts, attracting women with different pregnancy complications, including PROM. The hospital serves a high volume of clients, with its maternity department admitting 11,000 women annually. The hospital also has well-established departments in obstetrics, microbiology, and pediatrics, among others. The MRRH microbiology and molecular laboratory is a level 3 accredited laboratory and participates in external quality control conducted by the Uganda National Health Laboratory Services and the American College of Pathologists.

Study variables

The dependent variable was cervical amniotic fluid bacterial colonization. The independent variables included the socio-demographic factors (age and referral status), obstetric characteristics (gestational age, gravidity, number of antenatal care visits, duration of liquor drainage, antibiotic use since drainage started, and features of clinical chorioamnionitis, fever, maternal tachycardia, fetal tachycardia, foul-smelling liquor, abdominal tenderness), and medical factors (underlying medical illnesses, including HIV/AIDS, diabetes mellitus, urinary tract infections, abnormal vaginal discharge, and obesity). We categorized gestational age as preterm (<37 weeks) or term and beyond (≥ 37 weeks).

Inclusion and exclusion criteria

We included pregnant women admitted at ≥24 weeks of gestation with PROM during the study period (from December 21, 2020, to June 12, 2021). The attending obstetrician or resident doctor in obstetrics made the clinical diagnosis of PROM. The diagnosis was based on the history of a pregnant woman at the age of 24, who presented with a history of watery vaginal discharge before the onset of labor, pooling of fluid in the posterior vaginal fornix, or a flow of fluid from the cervical os either at rest or during coughing, as revealed by a sterile speculum examination.

We excluded patients who did not provide written consent and those with rupture of membranes before 24 weeks of gestation.

Sample size and sampling

Sample size estimation for this study was performed using Kish Leslie's formula for cross-sectional surveys [12]. The assumptions considered were a presumed proportion of cervical amniotic fluid colonization at 0.3, a desired margin of error of 5% at a 95% confidence level, and a source population of 240 women. Through a review of maternity registers, it was determined that the hospital admits approximately 40 women with PROM per month, leading to an estimated source population of 240 participants over a six-month period. Considering a 10% non-response rate, the final calculated sample size was determined to be 144 women. The participants were selected using consecutive sampling, and enrolment happened at all times, day or night, on each day of the study period.

Collection of data and endocervical samples

Each participant gave written informed consent. We used an interviewer-administered, pretested questionnaire to obtain data on participants’ demographics and medical and obstetric factors. Two sterile, individually packed endocervical samples were then collected by rotating the swab through 360° in the endocervical canal (except for every 10th participant, where two other samples were picked for analysis in another laboratory for quality control) and were labeled with unique participant numbers. The samples were delivered to the laboratory with a unique study identification number without any other clinical information.

Sample testing by culture

The endocervical sample was inoculated onto 5% sheep blood agar, MacConkey agar, Mannitol salt agar, and modified Thayer martin agar to isolate aerobic bacteria. The inoculated media was incubated at 37 °C aerobically for 24-72 hours. Modified Thayer martin agar plates were incubated in a humidified atmosphere with 5% carbon dioxide. Identification of the cultured isolate was done by conventional phenotypic and biochemical methods, which included catalase, coagulase, and DNA-ase for *S. aureus* (which produces positive catalase, coagulase, and DNA-ase tests) and urease, citrate utilization, oxidase, and triple sugar iron for identification and differentiation of Gram-negative bacilli.

Antimicrobial susceptibility testing was performed using the Kirby-Bauer disc diffusion method. The medium for fastidious organisms was chocolate agar, which was incubated in carbon dioxide. For non-fastidious organisms, we used Muller-Hinton Agar (MHA), incubated aerobically at 37 °C. The inoculum density required for susceptibility testing was 0.5% McFarland. The choice of antibiotic discs was based on the type of organism(s) cultured. The following antimicrobial agents were employed: ceftriaxone (30 μg), ciprofloxacin (5 μg), amoxicillin (10 μg), oxacillin (10 μg) and erythromycin (15 μg), gentamycin (10 μg), amoxicillin/clavulanate (10 μg), cefixime (10 μg), cefuroxime (10 μg), azithromycin (10 μg), and doxycycline (10 μg) were used for susceptibility testing.

Sample testing by PCR

The presence of the 16S rRNA gene (16SrDNA) was established by PCR amplification of genomic DNA using the following set of primers: Forward (AGAGTTTGATCMTGGCTCAG) and reverse (GGACTACCAGGGTATCTAATCCTGTT) primers that amplify the 16S rRNA gene were added to the two-times (2×) master mix containing standard buffer, dNTPs, Taq polymerase (M0486S) and nuclease-free water as follows: 12.5 µL of the 2× master mix; 1.0 µL forward (25 µM), 1.0 µL reverse (25 µM), 5 µL DNA template. RNAase-free H2O was used to make up the final reaction volume of 25 µL.

PCR conditions were as follows: initial denaturation at 95 °C for 60 seconds, followed by 35 cycles at 95 °C for 10 seconds, 54 °C for 10 seconds, and 72 °C for 50 seconds with a final extension of 72 °C for 5 minutes [1,2]. Gel electrophoresis was performed using a 1.2% agarose gel containing Safe View DNA stain, 6× loading dye (Thermo Scientific #R0611), and 500 bp molecular weight marker (NEB-Biolabs #N3231L) for 45 minutes at 120 V. PCR amplicons were visualized using the Gene-Flash Trans-illuminator.

Data management and analysis

Data were entered into Redcap and exported to STATA version 15 (StataCorp, Texas, USA) for analysis. The prevalence of cervical amniotic fluid bacterial colonization was determined by calculating the proportion of women who tested positive for either a positive culture or PCR, divided by the total number of women enrolled in the study, and expressed as a percentage.

The distribution of bacterial isolates was visually represented using a bar graph, where each isolate was depicted alongside its corresponding total count and percentage relative to the overall isolates. In terms of antibiotic susceptibility, a tabular format was utilized to present the susceptibility patterns of the isolates, indicating the frequencies and percentages for each bacterial isolate.

We used univariable and multivariable logistic regression to identify factors associated with cervical amniotic fluid bacterial colonization. Odds ratios and their corresponding 95% confidence intervals were reported at univariable analysis. Variables with a p-value <0.2 at univariable analysis and biologically plausible factors (gestational age, duration of PROM, presence of urinary tract infection, history of abnormal vaginal discharge, and presence of abdominal tenderness) were included in the multivariable analysis model. Factors with a p-value <0.05 at multivariable analysis were taken as statistically significant.

## Results

Baseline socio-demographic and obstetric characteristics of study participants

We enrolled 144 participants with a mean age of 26.5±6.2 years; most participants (75%) were in the 20-30-year age category (Table 1). The women with and those without cervical amniotic fluid bacterial colonization did not differ significantly in the distribution of most of the baseline characteristics except for gravidity (p=0.009) and referral status (p=0.038). Primigravidae women had more bacterial colonization (54.9%) than multigravidas (45.1%). A higher proportion of women who were referred to the hospital (70.6%) had cervical amniotic fluid bacterial colonization compared to those who were not referred (52.7%). There was no response, as all the eligible women who were approached accepted to participate in the study.

**Table 1:** Socio-demographic, obstetric and clinical characteristics of women with premature rupture of membranes at Mbarara Regional Referral Hospital, December 2020–June 2021 (N=144) SD: standard deviation; PROM: premature rupture of membranes, HIV: human immunodeficiency virus, ANC: antenatal care

Variables	Category	Bacterial colonization			p-value
		Overall, N=144(%)	Yes, N=51(%)	No, N=93 (%)	
Age (years)	20–30	108 (75.0)	39 (76.5)	69 (74.1)	0.265
	<20	18 (12.5)	9 (17.6)	9 (9.7)	
	35+	18 (12.5)	3 (5.9)	15 (16.1)	0.117
Residence	Rural	88 (61.1)	33 (64.7)	55 (59.1)	0.513
	Urban	56 (38.9)	18 (35.3)	38 (40.9)	
Marital status	Married	129 (89.6)	44 (86.7)	85 (91.4)	0.340
	Not married	15 (10.4)	7 (13.7)	8 (8.6)	
Referred	No	59 (41.0)	15 (29.4)	44 (47.3)	0.038
	Yes	85 (59.0)	36 (70.6)	55 (52.7)	
Body mass index	Normal	41 (28.7)	10 (19.6)	31 (33.7)	0.145
	Overweight	60 (42.0)	23 (45.1)	37 (40.2)	
	Obese	42 (29.4)	18 (35.3)	24 (26.1)	0.078
Urinary tract infection	No	93 (64.6)	35 (68.6)	58 (62.4)	0.453
	Yes	51 (35.4)	16 (31.3)	35 (37.6)	
Abnormal vaginal discharge	No	131 (91.0)	45 (88.2)	86 (92.5)	0.400
	Yes	13 (9.0)	6 (11.8)	7 (7.5)	
HIV status	Negative	127 (88.2)	46 (90.2)	81 (87.1)	0.583
	Positive	17 (11.8)	5 (9.8)	12 (12.9)	
Gravidity	Multigravida	86 (59.7)	23 (45.1)	63 (67.7)	0.009
	Primigravida	58 (40.3)	28 (54.9)	30 (32.3)	
Gestational age	<37 weeks	53 (36.8)	17 (23.3)	36 (38.7)	0.523
	≥37 weeks	91 (63.2)	34 (66.7)	57 (61.3)	
ANC attendance	≤4	87 (60.4)	30 (58.8)	57 (61.3)	0.772
	>4	57 (39.6)	21 (41.2)	36 (38.7)	
Duration of PROM	<12 hours	84 (58.3)	27 (53.0)	57 (61.3)	0.332
	≥12hours	60 (41.7)	24 (47.1)	36 (38.7)	
Presence of foul-smelling liquor	No	121 (84.0)	40 (78.4)	81 (87.1)	0.179
	Yes	23 (16.0)	11 (21.6)	12 (12.9)	
Abdominal tenderness	No	137 (95.1)	49 (96.1)	88 (94.6)	0.699
	Yes	7 (4.9)	2 (3.9)	5 (5.4)	
Fever	No	139 (96.5))	50 (98.0)	89 (95.7)	0.474
	Yes	5 (3.5)	1 (2.0)	4 (4.3)	
Maternal tachycardia	No	115 (79.9)	43 (84.3)	72 (77.4)	0.326
	Yes	29 (20.1)	8 (15.7)	21 (22.6)	
Antibiotic use since draining started	Yes	29 (20.1)	12 (23.5)	17 (18.3)	0.454
	No	115 (79.9)	39 (76.5)	76 (81.7)	

Prevalence of cervical amniotic fluid bacterial colonization

Among the 144 women with PROM at gestational age ≥24 weeks, a total of 51 participants (44 identified by culture and an additional 7 by DNA PCR) were found to have bacterial colonization, resulting in a prevalence of 35.4% (95% confidence interval [CI]: 28.0-43.7).

Bacterial isolates from cervical amniotic fluid

**Figure 1:**
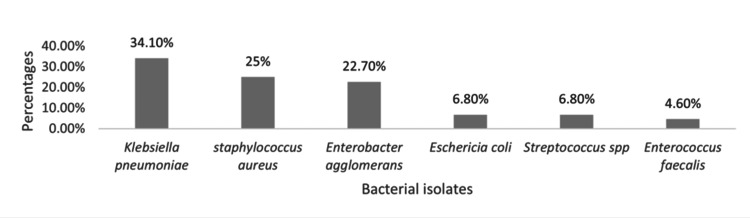
Bacterial isolates by culture in cervical amniotic fluid of women with premature rupture of membranes at Mbarara Regional Referral Hospital, December 2020–June 2021 (n=44)

A total of six different bacteria were isolated, with each sample containing only one bacterial isolate. Among the 44 isolates, the majority (n=28; 63.6%) were Gram-negative. *K. pneumoniae* was the most frequent isolate (n=15; 34.1%), followed by *S. aureus* (n=11; 25.0%), *Enterobacter agglomerans* (n=10; 22.7%), and the remaining isolates consisted of *E. coli* (6.8%), Streptococcus spp. (6.8%), and *Enterococcus faecalis* (4.6%) (Figure 1).

Antibiotic susceptibility patterns for the bacterial isolates

Of the 44 isolated bacteria, a notable majority (88.6%) were sensitive to ciprofloxacin, cefuroxime (75%), and ceftriaxone (72.7%). However, all the isolated bacteria were resistant to ampicillin, while a significant portion were resistant to amoxicillin (72.7%) and azithromycin (54.5%), as shown in Table 2.

**Table 2:** Antibiotic susceptibility patterns of the bacterial isolates in the cervical amniotic fluid of women with premature rupture of membranes at Mbarara Regional Referral Hospital, December 2020 to June 2021, (n=44) AMP: ampicillin; AMO: amoxicillin; AMOCLAV: amoxicillin/clavulanic acid; AZITHRO: azithromycin; OXAC: oxacillin; CEFIX: cefixime; CEFTRI: ceftriaxone; CIPRO: ciprofloxacin; DOXY: doxycycline; GENTA: gentamycin; CEFUROX: cefuroxime; ERYTH: erythromycin; S: sensitive; R: resistant.*Number of isolates are less than 5 (interpret the percentages in these cells with caution as the extremely small sample sizes make the percentages unstable).

Antibiotics		Bacterial isolates						
		*S. aureus* (n=11), n (%)	*Strep.* (n=3), n (%)	*E. faecalis* (n=2), n (%)	*Klebsiella* (n=15), n (%)	*E. coli* (n=3), n (%)	*E. agglomerans* (n=10), n (%)	Total (n=44), n (%)
AMO	S	5 (45.5)	1 (33.3)*	0 (0.0)*	2 (13.3)	1 (33.3)*	3 (30.0)	12 (27.3)
	R	6 (54.6)	2 (66.7)	2 (100)	13 (86.7)	2 (66.7)	7 (70.0)	32 (72.7)
AMP	S	0 (0.0)	0 (0.0)*	0 (0.0)*	0 (0.0)	0 (0.0)*	0 (0.0)	0 (0.0)
	R	11 (100)	3 (100)	2 (100)	15 (100)	3 (100)	10 (100)	44 (100)
AMOCLAV	S	9 (81.8)	2 (66.7)*	0 (0.0)*	9 (60.0)	1 (33.3)*	5 (50.0)	26 (59.1)
	R	2 (18.2)	1 (33.3)	2 (100)	6 (40.0)	2 (66.7)	5 (50.0)	18 (40.9)
OXAC	S	8 (72.7)	2 (66.7)*	2 (100)*	0 (0.0)*	0 (0.0)*	0 (0.0)*	12 (75.0)
	R	3 (27.3)	1 (33.3)	0 (0.0)	0 (0.0)*	0 (0.0)*	0 (0.0)*	4 (25.0)
AZITHRO	S	5 (45.5)	3 (100)*	1 (50.0)*	8 (53.3)	1 (33.3)*	2 (20.0)	20 (45.5)
	R	6 (54.5)	0 (0.0)	1 (50.0)	7 (46.7)	2 (66.7)	8 (80.0)	24 (54.5)
ERYTH	S	2 (18.2)	0 (0.0)*	0 (0.0)*	0 (0.0)*	0 (0.0)*	0 (0.0)*	2 (12.5)
	R	9 (81.8)	3 (100)	2 (100.0)	0 (0.0)*	0 (0.0)*	0 (0.0)*	14 (87.5)
CEFIX	S	8 (72.7)	2 (66.7)*	0 (0.0)*	6 (40.0)	1 (33.3)*	8 (80.0)	25 (56.8)
	R	3 (27.3)	1 (33.3)	2 (100)	9 (60.0)	2 (66.7)	2 (20.0)	19 (43.2)
CEFTRI	S	10 (90.9)	3 (100)*	1 (50.0)*	9 (60.0)	2 (66.7)*	7 (70.0)	32 (72.7)
	R	1 (9.1)	0 (0.0)	1 (50.0)	6 (40.0)	1 (33.3)	3 (30.0)	12 (27.3)
CEFUROX	S	10 (90.9)	3 (100)*	2 (100)*	10 (66.7)	2 (66.7)*	6 (60.0)	33 (75.0)
	R	1 (9.1)	0 (0.0)	0 (0.0)	5 (33.3)	1 (33.3)	4 (40.0)	11 (25.0)
CIPRO	S	9 (81.8)	3 (100)*	2 (100)*	14 (93.3)	3 (100)*	8 (80.0)	39 (88.6)
	R	2 (18.2)	0 (0.0)	0 (0.0)	1 (7.1)	0 (0.0)	2 (20.0)	5 (11.4)
GENTA	S	6 (54.5))	3 (100)*	1 (50.0)*	10 (66.7)	1 (33.3)*	6 (60.0)	27 (61.4))
	R	5 (45.5)	0 (0.0)	1 (50.0)	5 (33.3)	2 (66.7)	4 (40.0)	1 7 (38.6)
DOXY	S	0 (0.0)	1 (33.3)*	0 (0.0)*	5 (33.3)	0 (0.0)*	4 (40.0)	10 (22.7)
	R	11 (100)	2 (66.7)	2 (100)	10 (66.7)	3 (100)	6 (60.0)	34 (77.3)

Factors associated with cervical amniotic fluid bacterial colonization

Being primigravida, obese, or being referred to were associated with bacterial colonization. Primigravidae were about 2.7 times more likely to have cervical amniotic fluid bacteria colonization as compared to multigravidas (aOR: 2.69, 95% CI: 1.07-6.71, p=0.035). Obese women had three times higher odds of cervical amniotic fluid bacteria colonization compared to those with a normal body mass index (aOR: 3.15, 95% CI: 1.10-9.11, p=0.024). Women who were referred had approximately 2.37 times higher odds of cervical amniotic fluid bacterial colonization than those who were not referred (aOR=2.37, 95%CI: 1.04-5.3, p=0.038) (Table 3).

**Table 3:** Factors associated with cervical amniotic fluid bacterial colonization among women with PROM at Mbarara Regional Referral Hospital, December 2020 to June 2021 cOR: crude odds ratio; Ref: reference group; CI: confidence Interval; aOR: adjusted odds ratio; PROM: premature rupture of membranes.

Variables	Category	Bacterial colonization (n=51), n (%)	cOR (95% CI)	p-value	aOR (95% CI)	p-value
Age (years)	20-34	39 (76.5)	Ref.		Ref.	
	<20	9 (17.6)	1.77 (0.65–4.83)	0.265	1.05 (0.24–3.14)	0.931
	35+	3 (5.9)	0.35 (0.10–1.30)	0.117	0.48 (0.12–1.93)	0.298
Gravidity	Multigravida	23 (45.1)	Ref.		Ref.	0.035
	Primigravida	28 (54.9)	2.56 (1.27–5.16)	0.009	2.69 (1.07–6.71)	
Body mass index	Normal	10 (19.6)	Ref.		Ref.	
	Overweight	23 (45.1)	1.93 (0.80–4.66)	0.145	1.62 (0.62–4.30)	0.323
	Obese (≥30)	18 (35.3)	2.33 (0.91–5.95)	0.078	3.15 (1.10–9.11)	0.034
Gestational age	≥37 weeks	17 (23.3)	Ref.		Ref.	0.779
	<37 weeks	34 (66.7)	0.79 (0.39–1.62)	0.523	0.89 (0.39–2.01)	
Duration of PROM	<12 hours	27 (53.0)	Ref.		Ref.	0.323
	≥12hours	24 (47.1)	1.41 (0.71–2.81)	0.332	1.49 (0.68–3.26)	
Presence of foul-smelling liquor	No	40 (78.4)	Ref.		Ref.	0.204
	Yes	11 (21.6)	1.86 (0.75–4.57)	0.179	2.05 (0.68–6.23)	
Urinary tract infection	No	35 (68.6)	Ref.		Ref.	0.141
	Yes	16 (31.3)	0.76 (0.37–1.56)	0.453	0.54 (0.24–1.23)	
History of abnormal vaginal discharge	No	45 (88.2)	Ref.		Ref.	0.288
	Yes	6 (11.8)	1.64 (0.52–5.17)	0.400	2.01 (0.56–7.95)	
Referred	No	15 (29.4)	Ref.		Ref.	0.038
	Yes	36 (70.6)	2.15 (1.04–4.46)	0.038	2.37 (1.04–5.37)	

## Discussion

This study found that 35.4% (95% confidence interval: 28.0-43.7) of women with PROM seeking care at MRRH had amniotic bacterial colonization. The most common bacteria isolated was *K. pneumoniae*, followed by *S. aureus*. There was good sensitivity to quinolones and cephalosporins and marked resistance to penicillin. Prime gravida, obesity, and referrals were associated with amniotic bacterial colonization. These findings highlight the need to periodically review and update guidelines for the prophylactic use of antibiotics in PROM management; revising treatment protocols and considering alternative antibiotics based on local resistance patterns could improve patient outcomes and prevent complications associated with ineffective antibiotic therapy. 

The prevalence of cervical amniotic bacterial colonization of 35.4% reported in the current study is consistent with findings from studies conducted at Mulago Hospital, Uganda (30% in 2017), and Wayne State University, USA (41% in 2015) [4,5]. However, a study at Stanford University, USA, in 2010 reported a higher prevalence of 50% [13]. The similarity of our findings with those at Wayne University could be attributed to the use of universal primers for PCR, which allows for the detection of a broad range of bacteria. In contrast, the higher prevalence at Stanford University may be due to the use of both universal primers and group-specific primers, enabling the detection of bacterial presence in a larger number of samples. The observed high prevalence of amniotic fluid bacterial colonization in our study is concerning, as previous research has linked such colonization to adverse pregnancy outcomes for both mothers and fetuses. For example, a study investigating the effects of amniotic fluid bacterial colonization on uterine activity and delivery outcomes found associations with poor cervical dilatation, response to oxytocin, and an increased risk of intrapartum infection [14]. Intrauterine infection following ascending vaginal colonization has also been implicated in the causation of preterm labor, preterm births, stillbirths, and early-onset neonatal sepsis, among other complications [15].

*K. pneumoniae*, the commonest isolate in our study, has also been found to predominate amniotic fluid colonization in PROM in studies conducted at a national referral hospital in Uganda [5] and in Nigeria [16]. The gastrointestinal tract is a major reservoir of *K. pneumoniae*. The proximity of the gastrointestinal and genital tracts poses a high risk for contamination, allowing the bacteria to ascend to the cervix. This ascending colonization can lead to inflammation and subsequent rupture of the amniotic membrane [17]. Some strains of *K. pneumoniae* lack the mannose content of the capsular polysaccharide that is recognized by the surface lectin of macrophages to mediate complement and antibody-independent phagocytosis. This makes them virulent and enables them to evade the body’s defense mechanisms [17].

*S. aureus*, a commonly found bacterium in the human skin microbiota, emerged as the second most prevalent isolate in our study. Similar findings have been reported in other studies conducted in India and a meta-analysis from China [11], indicating its predominance in amniotic fluid colonization during PROM. As a resident flora on the skin, *S. aureus* can easily migrate to the genital tract and subsequently ascend to the cervix. This ascent can lead to infection and inflammation of the amniotic membranes, ultimately resulting in PROM [17,18]. Of particular significance, *S. aureus* produces α-toxin, which facilitates the formation of biofilms. Biofilm formation serves as a protective mechanism against dehydration and immune factors such as neutrophils and macrophages [19].

In contrast to studies conducted in Canada, Australia, and America where GBS was identified as the most common organism colonizing amniotic fluid in PROM, it was not the case in our study. This discrepancy may be attributed to global variations in GBS colonization among pregnant women. A systematic review encompassing 85 countries revealed significant regional variation, with higher prevalence observed in America and Canada (25%), and lower incidence in East Africa (18%) [20]. These regional disparities can be influenced by factors such as temperature variations, genetic factors, and differences in population demographics [21]. Additionally, socioeconomic factors and variations in natural immunity within different populations can play a significant role. It is worth noting that a considerable number of women in our study were referred from other healthcare facilities. As prophylaxis against GBS is part of the clinical guidelines [10], these referred cases may have already received prophylactic treatment, which could explain the lower prevalence of GBS in this study.

The bacteria isolated in our study exhibited significant resistance to the antibiotics recommended by the World Health Organization (WHO) and adopted by the Ministry of Health in Uganda for prophylaxis in PROM. All isolates were resistant to ampicillin, and the majority showed resistance to erythromycin, amoxicillin, and azithromycin. It is noteworthy that these guidelines were established based on recommendations from the ORACLE study, conducted over 20 years ago, which did not specifically focus on bacterial colonization of the female genital tract or antibiotic resistance [22]. The resistance to ampicillin observed in our study aligns with findings from other studies conducted in Nigeria and Uganda [5,16]. This resistance pattern may be attributed to the overuse of these antibiotics, as Penicillin is commonly prescribed for various bacterial infections. The overuse of antibiotics directly contributes to the emergence of drug-resistant bacterial strains, and these resistance genes can be inherited or acquired and transferred among different species of bacteria. Additionally, antibiotics eliminate drug-sensitive competitors, providing a selective advantage for resistant bacteria to proliferate through natural selection [23]. The outdated nature of the guidelines, coupled with the alarming rates of resistance observed in this study, emphasize the importance of updating and tailoring treatment protocols to address the evolving antibiotic resistance landscape and improve patient outcomes.

The sensitivity of the isolated bacteria to certain cephalosporins was notable, with ceftriaxone demonstrating a sensitivity rate of 72.7% and the less commonly prescribed second-generation cefuroxime exhibiting a sensitivity rate of 75%. Similar findings have been reported in a meta-analysis conducted in China as well as studies conducted in Nigeria and Uganda [5,11,16]. This could be attributed to the stable β-lactam ring present in cephalosporins, which confers resistance to the action of beta-lactamases, enzymes that can inactivate certain antibiotics [24]. Overall, the highest sensitivity was observed for ciprofloxacin, at 88.6%, which aligns with findings from Nigeria, where it reached 96.3% [16]. This could be attributed to ciprofloxacin having experienced a period of high resistance in the past, leading to its exclusion from many treatment protocols. As a result, its usage has been limited in recent years [25]. These findings further highlight the importance of selecting appropriate antibiotics for management of PROM based on their sensitivity profiles and considering the local resistance patterns. Continued surveillance of antimicrobial resistance could inform prescribing practices and ensure effective treatment outcomes.

Primigravidae were more likely to have cervical amniotic fluid bacteria colonization as compared to multigravidas. Primigravidae may be at a greater risk of bacterial colonization than multigravidas due to their relatively limited interactions with the healthcare system and the potential lack of exposure to medications that reduce bacterial colonization [26]. Given this increased vulnerability, primigravidae should be prioritized in healthcare settings to avoid consequences associated with cervical amniotic fluid bacterial colonization in PROM.

Our findings revealed that obese women were more likely to exhibit cervical amniotic fluid bacterial colonization compared to women with a normal BMI. This observation aligns with previous studies that have demonstrated a link between obesity and bacterial colonization in the female genital tract [27]. Notably, a significant proportion of bacteria colonizing the amniotic fluid in cases of PROM ascend from the genital tract. The association between obesity and increased bacterial colonization in the genital tract may be attributed to several factors. First, obesity can lead to poor genital hygiene due to excessive sweating and genital perspiration, creating an environment conducive to bacterial growth [27]. Furthermore, obese women often have higher estrogen levels resulting from peripheral aromatization, which promotes the maturation, proliferation, and accumulation of glycogen in vaginal epithelial cells. This glycogen serves as a favorable culture medium for bacterial growth [28]. As reported elsewhere, it is crucial to prioritize the care of obese mothers by promoting good hygiene practices and implementing dietary and physical activity adjustments to mitigate the risk of cervical amniotic fluid bacterial colonization in cases of PROM [29].

Women who were referred had higher odds of having cervical amniotic fluid bacteria than those who were not referred. One plausible explanation for this observation is that a significant proportion of women with PROM in our study were referred from lower-level healthcare facilities where protocols for the accurate diagnosis and management of PROM may be lacking. Consequently, these mothers may have undergone multiple vaginal examinations and experienced a prolonged latency period before their presentation, primarily due to the challenges associated with referral transportation in our setting [30]. However, the effect of the number of vaginal examinations was not evaluated in this study. This may have resulted in this residual confounding from this unstudied factor. Of the 51 participants who had cervical amniotic fluid bacterial colonization, only 15 were not referred, and such a small sample could also accentuate the association.

What is already known on this topic?

Bacterial colonization is implicated in the causation and complications of PROM. Streptococcus is the most common organism that colonizes the genital tract of pregnant women and is found in the amniotic fluid of women with PROM. Therefore, prophylaxis in PROM targets the changing amniotic fluid bacterial colonization patterns and antibiotic susceptibilities in women with PROM in different regions.

What this study adds?

In our setting, the prevalence of bacterial colonization in amniotic fluid is high among women with PROM. The commonest bacteria colonizing amniotic fluid are *K. pneumoniae* and *S. aureus*, as opposed to Group B streptococci, for which we give prophylaxis. Women with PROM can potentially replace prophylaxis with cefuroxime due to its 100% resistance to ampicillin and good sensitivity.

Limitations

Our study had some limitations that should be considered. First, we could not conduct gene sequencing on the PCR-positive samples, which restricted our ability to identify and characterize specific bacterial isolates present in the samples. This information could have provided valuable insights into the microbial composition and potential virulence factors associated with cervical amniotic fluid bacterial colonization. Second, our sample size was also not powered to determine any causal inference, did not adjust for all sociodemographic characteristics, and our study was conducted at a single site, which may limit the generalizability of our findings to healthcare settings different from ours. Despite these limitations, our study benefited from highly sensitive PCR diagnostic testing, which improved the accuracy of detecting bacterial colonization in addition to traditional culture methods. This enhanced sensitivity strengthens the validity of our findings regarding the prevalence of bacterial colonization in women with PROM. We were also able to obtain important descriptive epidemiological information on associations between cervical amniotic fluid and bacterial colonization.

## Conclusions

Approximately 35.4% of the women in this study had cervical amniotic fluid bacterial colonization. Alarmingly, all the bacterial isolates demonstrated resistance to ampicillin, the recommended first-line treatment according to the Ministry of Health guidelines for PROM. This high prevalence of bacterial colonization, coupled with the resistance patterns observed, underscores the urgency of reviewing the current guidelines for the prophylactic use of ampicillin in PROM in our setting. Future longitudinal studies should assess the impact of cervical amniotic fluid bacterial colonization on maternal and perinatal outcomes to develop evidence-based management strategies that optimize clinical outcomes of women with PROM in the region and similar low-resource settings.
